# Associations between neighbourhood environmental characteristics and obesity and related behaviours among adult New Zealanders

**DOI:** 10.1186/1471-2458-14-553

**Published:** 2014-06-04

**Authors:** Amber L Pearson, Graham Bentham, Peter Day, Simon Kingham

**Affiliations:** 1 Department of Public Health, University of Otago - Wellington, 23A Mein Street, Wellington 6242, New Zealand; 2 School of Environmental Sciences, University of East Anglia, Norwich NR4 7TJ, UK; 3 Department of Geography, University of Canterbury, GeoHealth Laboratory, Private Box 1400, Christchurch 8140, New Zealand

**Keywords:** Obesity, Built environment, Neighbourhood, New Zealand

## Abstract

**Background:**

The prevalence of adult obesity is escalating in most wealthy and middle income countries. Due to the magnitude of this issue, research and interventions at the individual-level abound. However, the limited success and high costs of such interventions has led to a growing recognition of the potential role of environmental factors in reducing obesity and promoting physical activity and healthy diets.

**Methods:**

This study utilised individual-level data from the 2006/7 New Zealand Health Survey on obesity, physical activity, diet and socio-economic variables linked to geographic information from other sources on potentially aetiologically-relevant environmental factors, based on the respondent’s residential address. We fitted logistic regression models for eight binary measures of weight or weight-related behaviours: 1) overweight; 2) obesity; 3) overweight + obesity; 4) active at least 30 minutes a day for 5+ days per week; 5) active <30 minutes per week; 6) walk 150 minutes + per week; 7) walk <30 minutes per week; and 8) consumption of 5+ fruits and vegetables per day. We included a range of independent environmental characteristics of interest in separate models.

**Results:**

We found that increased neighbourhood deprivation and decreased access to neighbourhood greenspace were both significantly associated with increased odds of overweight and/or obesity. The results for weight-related behaviours indicate that meeting the recommended level of physical activity per week was associated with urban/rural status, with higher activity in the more rural areas and a surprising tendency for less activity among those living in areas with higher levels of active travel to work. Increased access to greenspace was associated with high levels of walking, while decreased access to greenspace was associated with low levels of walking. There was also a significant trend for low levels of walking to be positively associated with neighbourhood deprivation. Results for adequate fruit and vegetable consumption show a significant urban/rural gradient, with more people meeting recommended levels in the more rural compared to more urban areas.

**Conclusion:**

Similar to findings from other international studies, these results highlight greenspace as an amenable environmental factor associated with obesity/overweight and also indicate the potential benefit of targeted health promotion in both urban and deprived areas in New Zealand.

## Background

In 2013, Mexico surpassed the United States with about one-third of its adults estimated to be obese [1]. However, the United States [2], the United Kingdom, New Zealand and other wealthy countries are not far behind and are on upward trajectories. The prevalence of adult obesity in New Zealand is high (28% in 2011/2) and rising (from 26% in 2006/7) [3], leading to escalating health care costs, especially for associated conditions such as Type II diabetes. At its most basic, obesity results from a positive balance between energy input from food and drink and energy output from basic metabolic processes and from physical activity, with excess calories being stored as body fat [4]. Due to the magnitude of this health issue and the myriad diseases related to obesity, research to understand behaviour, genetics, surgery or drug therapies to reduce or prevent obesity have been widespread [5-8]. Most interventions have focused on individuals, targeting behaviours related to diet and physical activity, or the use of anti-obesity drugs, or, in extreme cases, bariatric surgery. The limited success and high costs of such interventions has led to a growing recognition of the importance of obesity at a population-level and the role of supply side interventions [9] and amenable environmental factors in obesity, particularly those that encourage higher levels of physical activity and healthy diets [10].

Evidence of individual-level risk factors for adult obesity include childhood obesity [11], income, education [12] and interactions in risk by age, sex and ethnicity [13] most likely related to varying behaviours and diet [9]. While behaviours and diet are important direct risk factors for obesity, it is the environments in which individuals reside which may play a large role in such risk factors. With the exception of neighbourhood deprivation, studies have had mixed results when using GIS-based measures to capture neighbourhood environmental variables which may promote or hinder the maintenance of a healthy weight for residents measured against both individual and population level obesity or weight-related behaviours [14,15]. These GIS-based exposure measures include proximity of supermarket, density or type of food outlet, neighbourhood ‘walkability’, landuse, greenspace, and population density. Outcome measures include BMI, obesity status, and weight-related behaviours such as fruit/vegetable consumption and physical activity. For example, findings are inconsistent in terms of the role of access to fresh fruits and vegetables on healthy weight. Accessibility of fruits and vegetables was positively associated with increased consumption in most, but not all, of the studies in the USA and Norway [16-19]. Studies found inconsistent associations between consumption and the establishment of food outlets in the United Kingdom [20,21]. In New Zealand, geographic access to supermarkets was better in deprived neighbourhoods than affluent neighbourhoods, but access was not associated with individuals’ vegetable intake [22]. A recent review of the evidence to support a link between access to greenspace and weight reported that almost 70% of studies found a positive or weak association between greenspace and obesity-related health indicators. However, the review concluded that findings varied by age of study participants, socioeconomic status and the geographic measure of greenspace used [23]. Another important obesity-related focus of neighbourhood-based research is the role of the built environment in promoting physical activity. Sallis et al. have put forward key findings and recommendations to modify these features in order to increase physical activity. Specifically, this report emphasizes proximity to recreation facilities and access to active transportation [10]. Internationally, the findings are much more consistent for neighbourhood socio-economic status or deprivation and weight-related outcomes. The Whitehall studies in the United Kingdom (UK) showed strong associations over time with BMI in women regardless of individual income, age, smoking status, alcohol intake, or physical activity level [24]. Similar results were found for both men and women in the Netherlands. Even after adjustment for education, age and sex the odds ratios of overweight increased significantly by increasing neighbourhood deprivation [25].

Despite much of the international evidence finding associations between neighbourhood characteristics and obesity, the current evidence-base in New Zealand for guiding the design of interventions in obesogenic environments is sparse. This study, therefore, aimed to understand the potential influence of neighbourhood environments on both unhealthy weight outcomes (overweight and obese) and weight-related behaviours (walking, physical activity levels and fruit and vegetable consumption). To our knowledge, this is the first study in New Zealand to evaluate the potential role of environmental characteristics in influencing individual-level obesity/overweight, adding to evidence from the USA, Australia, Canada and Europe [15].

## Methods

The study is based on individual-level data from the 2006/7 New Zealand Health Survey (NZHS) on obesity, physical activity including walking and diet [26] linked to geographic information from other sources on potentially aetiologically-relevant environmental factors, based on the respondent’s residential address at the time of the survey.

### Health data

The 2006/07 NZHS was conducted from October 2006 to November 2007. Data were collected for 12,488 adults aged 15 years and over (response rate of 68%) [26]. The NZHS is a key component of national health monitoring by the New Zealand Ministry of Health and is designed to be a nationally-representative sample of New Zealanders. This survey used a multi-stage, stratified, probability proportionate to size sample design, with increased sampling of some ethnic groups. A full description of the sampling design is available online^a^. In brief, small geographic areas (meshblocks) were randomly selected and interviewers began at a random point in each meshblock and selected every *k*th house for enrolment of one adult aged 15 years and over. Interviews were conducted in participants’ homes, at a time to suit participants. Height, weight and waist measurements were taken using professional weighing scales, a portable stadiometer, and an anthropometric measuring tape.

For our research purposes, the NZHS health and behavioural end-points of interest were: 1) Body Mass Index (BMI) values and as internationally recognised categories of overweight; 2) obesity; 3) and either overweight or obesity; 4) Whether the individual meets the recommendation of at least 30 minutes per day of moderate/vigorous physical activity on at least 5 days in a week [27]; 5) Whether the individual had 30 minutes or less per week of moderate/vigorous physical activity as a measure of sedentary behaviour [26]; 6) Whether the individual walks at least 150 minutes in a week, as walking has long been recognised as an important low to moderate form of physical activity [28]; 7) Whether the individual walks <30 minutes per week as a measure of sedentary behaviour [26]; and 8) Whether the individual consumes 5 or more portions of fruit and vegetables per day as recommended by the United States Department of Agriculture and other agencies [29].

### Neighbourhood environmental characteristics data

We compiled geographic data for a number of neighbourhood characteristics, posited as important environmental influences in previous research (Table 1). Drawing on the framework outlined by Sallis *et al.*[10], the environmental characteristics in this study included aspects of the built environment (e.g., food outlets [30] and green space [31]) and the social/cultural environment (e.g., area-level deprivation [3]). The number of variables was limited and all continuous variables were converted to quintiles (1 = best access and 5 = worst access) because of a New Zealand Ministry of Health requirement for maintaining confidentiality. The selected neighbourhood variables were then linked, by the Ministry of Health, to the individual-level NZHS responses by the residential address at the time of the survey and then addresses were removed for anonymity prior to analyses. Some variables were measured at the meshblock level (average 2006 population ~ 100), which is the smallest unit of aggregation in New Zealand. Others were measured at the census area unit (CAU) level (average 2006 population ~2500), which is the next larger unit of aggregation and usefully approximates a neighbourhood in urban settings.

**Table 1:** Sources and descriptions of neighbourhood environmental characteristics data

**Characteristic**	**Description**	**Source (Year)**	Descriptive statistics*
Urban/rural category	CAU ranking 1 to 4, where 1 = most urban	Statistics New Zealand (2006)	Min = 1, 25^th^ percentile = 1, Mean = 1.4, Median = 1, 75^th^ percentile = 1, Max = 4
Area-level deprivation (NZDep)	NZDep 2006 quintiles for meshblocks, where 1 = least deprived	Salmond (2006) [32]	Min = 1, 25^th^ percentile = 2, Mean = 3.2, Median = 3, 75^th^ percentile = 5, Max = 5
Accessibility of useable greenspace	Proportion of meshblock consisting of useable greenspace, as qunitles where 1 = best access	Richardson (2005) [33]	Min = 1, 25^th^ percentile = 2, Mean = 2.9, Median = 3, 75^th^ percentile =4, Max = 5
Accessibility of food outlets	Distance from meshblock population-weighted centroid to nearest outlet (supermarkets, fast-food outlets, convenience stores), as quintiles where 1 = nearest	Territorial Authorities (2005) [34]	Min =1, 25^th^ percentile = 2, Mean = 2.8, Median = 3, 75^th^ percentile = 4, Max = 5
Accessibility of sports/leisure facilities	Distance from population-weighted centroid of meshblock to nearest gym, pool, karate, recreation centre, as quintiles where 1 = nearest (excludes biking/hiking trails)	ACC Pool Safety (2005) [35]	Min = 1, 25^th^ percentile = 2, Mean = 2.8, Median = 3, 75^th^ percentile = 4, Max = 5
Percentage active transport to work	Proportion of CAU adult residents who walk, bus or cycle to work, as quintiles, where 1 = least	Statistics New Zealand (2006)	Min = 1, 25^th^ percentile = 2, Mean = 3, Median = 3, 75^th^ percentile = 4, Max = 5

*Calculated only for areas where health survey participants resided.

### Potential confounder data

The NZHS also provides data at the individual-level on potential confounders/effect modifiers, including age, sex, ethnicity, Economic Living Standard Index [36], individual-level deprivation (NZiDep) [37], highest educational qualification, household composition, smoking status and alcohol consumption.

### Statistical analyses

Separate logistic regression models were fitted for eight binary dependent variables: 1) overweight; 2) obesity; 3) overweight + obesity; 4) active at least 30 minutes a day for 5+ days per week; 5) active <30 minutes per week; 6) walk 150 minutes + per week; 7) walk <30 minutes per week; and 8) consumption of 5+ fruits and vegetables per day. Each model was first fitted unadjusted (i.e., each neighbourhood environmental factor one at a time for each of the dependent variables). Next, each model was fitted adjusted for individual-level confounders. Last, models were fitted for each dependent variable including all environmental factors as independent variables and adjusted for individual-level characteristics. We included the independent environmental characteristics of interest (quintiles) as continuous variables to provide tests of trend and as discrete categories for which adjusted Odds Ratios (ORs) and 95% confidence intervals were calculated. All models are fitted using Stata v11 (College Station, TX, USA) with adjustment for the complex sample design of the NZHS, which produced cluster robust estimates.

## Results

The majority of respondents were classed as either overweight or obese (65%) and the percentage of overweight/obese males was higher than females (70% and 60% respectively) (Table 2). Younger respondents had lower levels of overweight and obesity. Among ethnic groups, over 90% of Pacific respondents were classed as overweight/obese and 68% classed as obese. The Asian respondents had the lowest levels of overweight/obesity, with only 11% classed as obese.

**Table 2:** Demographic characteristics of respondents, by weight status

	**n**	**% overweight**	**% obese**	**% overweight or obese**
All	12488	37.1	27.5	64.6
Females	7215	31.3	27.9	59.2
Males	5273	43.1	27.1	70.2
Age 15-44	6320	33.6	23.9	57.5
Age 45-64	3808	39.2	32.8	72.0
Age 65+	2360	43.9	27.7	71.6
Māori	3160	31.9	45.0	76.9
Pacific	1033	23.1	67.7	90.8
European	8519	39.1	25.5	64.6
Asian	1513	31.5	11.0	42.5
Other	87	31.0	16.4	47.4

The results of our fully-adjusted regression analyses (Model 3), where the ORs represent tests of overall trends, indicate that overweight, obesity and overweight + obesity status were each associated with increased deprivation and lower access to greenspace (Table 3).

**Table 3:** Associations (tests of trend) between overweight, obesity, overweight + obesity and environmental factors

	**MODEL 1**			**MODEL 2**			**MODEL 3**		
	***(Run separately for each environmental factor)***			***(Run separately for each environmental factor)***			***(All environmental factors included)***		
	**Unadjusted**			**Adjusted individual factors**			**Adjusted individual factors and environmental factors**		
	**OR**	**95% CI**	**p-value**	**OR**	**95% CI**	**p-value**	**OR**	**95% CI**	**p-value**
**Overweight**									
Urban/rural	**1.11**	**1.03,1.19**	**0.004**	1.01	0.94,1.09	0.735	1.01	0.89,1.15	0.843
NZdep	1.01	0.96,1.06	0.688	**1.05**	**1.00,1.11**	**0.043**	**1.06**	**1.00,1.12**	**0.034**
Greenspace	**1.08**	**1.03,1.13**	**0.001**	1.04	1.00,1.09	0.073	**1.05**	**1.00,1.11**	**0.041**
Foodshop	**1.06**	**1.01,1.10**	**0.017**	1.00	0.95,1.05	0.981	0.99	0.93,1.07	0.880
Gym/pool	**1.10**	**1.06,1.15**	**0.000**	1.02	0.98,1.07	0.374	1.02	0.95,1.09	0.533
Active travel	**0.93**	**0.89,0.97**	**0.001**	0.98	0.93,1.02	0.282	0.97	0.92,1.03	0.322
**Obesity**									
Urban/rural	**1.16**	**1.08,1.25**	**<0.001**	1.08	1.00,1.17	0.060	1.07	0.93,1.22	0.361
NZdep	**1.23**	**1.16,1.30**	**<0.001**	**1.13**	**1.07,1.20**	**<0.001**	**1.11**	**1.05,1.18**	**0.001**
Greenspace	**1.14**	**1.09,1.20**	**<0.001**	**1.09**	**1.04,1.15**	**0.001**	**1.08**	**1.18,1.15**	**0.007**
Foodshop	1.02	0.97,1.07	0.523	0.98	0.93,1.03	0.429	0.94	0.88,1.02	0.137
Gym/pool	**1.17**	**1.11,1.23**	**<0.001**	1.06	1.00,1.11	0.042	1.06	0.98,1.15	0.143
Active travel	**0.89**	**0.85,0.94**	**<0.001**	0.97	0.92,1.02	0.217	0.97	0.91,1.03	0.314
**Overweight + obesity**									
Urban/rural	**1.13**	**1.06,1.20**	**<0.001**	1.08	1.00,1.17	0.060	1.04	0.92,1.17	0.525
NZdep	**1.10**	**1.05,1.16**	**<0.001**	**1.13**	**1.07,1.20**	**<0.001**	**1.08**	**1.02,1.13**	**0.004**
Greenspace	**1.10**	**1.06,1.15**	**<0.001**	**1.07**	**1.02,1.11**	**0.003**	**1.07**	**1.01,1.22**	**0.005**
Foodshop	1.04	1.00,1.08	0.069	0.98	0.93,1.03	0.429	0.97	0.91,1.03	0.320
Gym/pool	**1.13**	**1.09,1.18**	**<0.001**	1.06	1.00,1.11	0.042	1.04	0.98,1.11	0.217
Active travel	**0.91**	**0.87,0.95**	**<0.001**	0.97	0.92,1.02	0.217	0.97	0.92,1.03	0.315

Note: Categories: Urban/rural: 1 = Main urban area; 2 = Secondary urban area; 3 = Minor urban area; and 4 = Rural area. Environmental variables: quintiles (1 = best access, 5 = worst access); Deprivation (NZdep) (1 = least deprived, 5 = most deprived).

All bolded values are statistically significant at the 0.05 level.

Table 4 include the results of regression analyses, where the ORs represent comparisons with the reference categories, for weight status outcomes. For the fully adjusted model which included overweight as the dependent variable, the highest deprivation category was significantly associated with increased odds of being overweight (OR = 1.34, p = 0.018) compared to the least deprived group. Similarly, overweight status was significantly associated with decreased access to greenspace for each access category, except category three, compared to the reference category (best access).

**Table 4:** Association between overweight, obesity, overweight+obesity and environmental factors adjusted for socio-demographic and other environmental factors

	**Category 1**	**Category 2**			**Category 3**			**Category 4**			**Category 5**			
		**OR**	**95% CI**	**p-value**	**OR**	**95% CI**	**p-value**	**OR**	**95% CI**	**p-value**	**OR**	**95% CI**	**p-value**	**p trend**
**Overweight**														
Urban/rural	*Reference*	0.82	0.64,1.05	0.113	0.99	0.70,1.42	0.970	1.16	0.77,1.73	0.478				0.843
NZdep	*Reference*	1.19	0.98,1.44	0.078	1.10	0.89,1.34	0.378	1.21	0.97,1.49	0.089	**1.34**	**1.05,1.72**	**0.018**	**0.034**
Greenspace	*Reference*	**1.38**	**1.24,1.68**	**0.001**	1.14	0.93,1.39	0.215	**1.32**	**1.08,1.63**	**0.008**	**1.34**	**1.04,1.73**	**0.022**	**0.041**
Foodshop	*Reference*	0.97	0.80,1.18	0.773	0.99	0.81,1.20	0.909	1.04	0.82,1.32	0.753	0.82	0.55,1.18	0.265	0.880
Gym/pool	*Reference*	0.98	0.81,1.18	0.805	1.01	0.83,1.24	0.892	1.13	0.86,1.47	0.338	1.07	0.79,1.44	0.678	0.533
Active travel	*Reference*	1.00	0.82,1.23	0.973	1.06	0.85,1.34	0.586	1.03	0.83,1.27	0.811	0.89	0.70,1.13	0.340	0.322
**Obesity**														
Urban/rural	*Reference*	1.13	0.86,1.48	0.385	1.30	0.87,1.96	0.204	1.09	0.71,1.69	0.688				0.361
NZdep	*Reference*	0.93	0.74,1.17	0.537	1.04	0.82,1.33	0.749	1.18	0.93,1.51	0.177	**1.56**	**1.20,2.04**	**0.001**	**0.001**
Greenspace	*Reference*	1.00	0.79,1.26	0.521	0.97	0.77,1.22	0.797	**1.30**	**1.02,1.65**	**0.032**	**1.42**	**1.08,1.87**	**0.012**	**0.007**
Foodshop	*Reference*	**0.75**	**0.61,0.94**	**0.010**	0.90	0.73,1.12	0.343	0.79	0.61,1.04	0.088	**0.67**	**0.45,0.99**	**0.046**	0.137
Gym/pool	*Reference*	0.91	0.74,1.12	0.375	1.23	0.98,1.55	0.073	0.94	0.68,1.30	0.704	1.18	0.83,1.68	0.365	0.143
Active travel	*Reference*	1.15	0.90,1.48	0.269	1.16	0.88,1.54	0.291	1.10	0.84,1.46	0.485	0.90	0.67,1.21	0.497	0.314
**Overweight+obesity**														
Urban/rural	*Reference*	0.93	0.74,1.18	0.575	1.13	0.82,1.56	0.460	1.15	0.80,1.65	0.463				0.525
NZdep	*Reference*	1.09	0.91,1.31	0.328	1.06	0.88,1.28	0.547	1.17	0.97,1.42	0.109	**1.43**	**1.14,1.78**	**0.002**	**0.004**
Greenspace	*Reference*	**1.23**	**1.03,1.47**	**0.023**	1.08	0.90,1.29	0.433	**1.34**	**1.11,1.61**	**0.002**	**1.39**	**1.10,1.75**	**0.006**	**0.005**
Foodshop	*Reference*	0.88	0.74,1.04	0.128	0.94	0.79,1.11	0.437	0.92	0.74,1.14	0.444	0.74	0.53,1.03	0.079	0.320
Gym/pool	*Reference*	0.96	0.81,1.14	0.675	1.11	0.93,1.33	0.247	1.07	0.83,1.37	0.598	1.11	0.84,1.48	0.455	0.217
Active travel	*Reference*	1.09	0.89,1.32	0.401	1.12	0.90,1.39	0.313	1.10	0.89,1.36	0.379	0.91	0.73,1.15	0.428	0.315

Note: Categories: Urban/rural: 1 = Main urban area; 2 = Secondary urban area; 3= Minor urban area; and 4 = Rural area.

Environmental variables: quintiles (1 = best access, 5 = worst access); Deprivation (NZdep) (1= least deprived, 5= most deprived).

All bolded values are statistically significant at the 0.05 level.

For the model which included obesity as the dependent variable, the highest deprivation category was also significantly associated with increased odds of being obese (OR = 1.56, p = 0.001) compared to the least deprived group. Also, the two lowest levels of access to greenspace were significantly associated with increased odds of being obese (OR = 1.30 and OR = 1.42, p < 0.033) compared to the highest level of access. The second and the highest levels of access to foodshops were significantly associated with decreased odds of being obese (ORs 0.75 or lower, p < 0.05) compared to the lowest level of access.

For the model which included overweight or obesity as the dependent variable, similar trends were observed for deprivation, where those living in the most deprived neighbourhoods had significantly increased of being overweight/obese. Decreased access to greenspace also exhibited increased risk in all but one level of access, compared to the best access level.

The results of our fully adjusted regression (Model 3) analyses for meeting the recommended level of physical activity per week, where the ORs represent tests of overall trends, only two environmental variables remain significantly associated: 1) urban/rural status, with substantially more people meeting recommended standards of physical activity in the more rural areas than in the more urban areas; and 2) a significant and surprising tendency for fewer people to meet the recommended standard in areas with higher levels of active travel to work (OR = 0.94, p = 0.012) (Table 5). Fully-adjusted results for low overall physical activity indicated no environmental variables were significant (Model 3). Results for walking behaviours diverge from those for overall physical activity. First, decreased access to greenspace was associated with lower odds of walking at least 150 minutes/week (OR = 0.93, p <0.001), while decreased access to greenspace was associated with increased odds of walking less than 30 minutes per week (OR = 1.09, p < 0.001). There was also a significant trend for low levels of walking to be positively associated with neighbourhood deprivation. Results for adequate fruit and vegetable consumption show a significant urban/rural gradient, with more people meeting recommended levels in the more rural compared to more urban areas. Adequate fruit and vegetable consumption was also significantly lower for those living in the most deprived neighbourhoods, but the test of trend was not statistically significant in the fully adjusted model.

**Table 5:** Associations (test of trend) between obesity-related behaviours and environmental factors

	**MODEL 1**			**MODEL 2**			**MODEL 3**		
	***(Run separately for each environmental factor)***			***(Run separately for each environmental factor)***			***(All environmental factors included)***		
	**Unadjusted**			**Adjusted individual factors**			**Adjusted individual factors and environmental factors**		
	**OR**	**95% CI**	**p-value**	**OR**	**95% CI**	**p-value**	**OR**	**95% CI**	**p-value**
**Active 5+ days**									
Urban/rural	**1.25**	**1.17,1.33**	**<0.001**	**1.24**	**1.16,1.32**	**<0.001**	**1.12**	**1.01,1.25**	**0.038**
NZdep	0.99	0.96,1.03	0.777	1.01	0.96,1.05	0.810	1.02	0.97,1.07	0.389
Greenspace	**1.07**	**1.03,1.11**	**0.001**	**1.05**	**1.01,1.10**	**0.007**	0.99	0.95,1.04	0.579
Foodshop	**1.09**	**1.05,1.13**	**<0.001**	**1.07**	**1.03,1.12**	**0.001**	1.02	0.96,1.08	0.399
Gym/pool	**1.13**	**1.09,1.18**	**<0.001**	**1.12**	**1.08,1.17**	**<0.001**	1.00	0.94,1.06	0.942
Active travel	**0.93**	**0.89,0.96**	**<0.001**	**0.94**	**0.90,0.97**	**<0.001**	**0.94**	**0.89,0.99**	**0.012**
**Active <30 mins/week**									
Urban/rural	**0.90**	**0.82,0.99**	**0.028**	0.94	0.85,1.03	0.195	1.06	0.87,1.29	0.548
NZdep	**1.18**	**1.12,1.24**	**<0.001**	**1.10**	**1.04,1.17**	**0.001**	1.07	1.00,1.14	0.051
Greenspace	0.97	0.91,1.02	0.239	0.99	0.93,1.05	0.718	1.02	0.95,1.09	0.473
Foodshop	**0.88**	**0.83,0.93**	**<0.001**	**0.93**	**0.88,0.99**	**0.028**	0.99	0.91,1.09	0.887
Gym/pool	**0.92**	**0.87,0.98**	**0.007**	**0.93**	**0.88,0.99**	**0.032**	0.96	0.88,1.05	0.350
Active travel	**1.07**	**1.02,1.13**	**0.011**	**1.06**	**1.00,1.12**	**0.047**	1.01	0.94,1.09	0.726
**Walk 150+ mins/week**									
Urban/rural	**1.12**	**1.04,1.20**	**0.002**	**1.13**	**1.05,1.21**	**0.001**	1.06	0.95,1.17	0.284
NZdep	1.02	0.98,1.06	0.358	1.03	0.98,1.07	0.235	1.03	0.98,1.09	0.208
Greenspace	0.97	0.94,1.01	0.190	0.97	0.93,1.01	0.133	**0.93**	**0.89,0.97**	**<0.001**
Foodshop	1.03	0.99,1.08	0.140	1.03	0.99,1.08	0.149	1.02	0.95,1.08	0.625
Gym/pool	**1.05**	**1.00,1.09**	**0.040**	**1.05**	**1.00,1.10**	**0.034**	1.00	0.93,1.07	0.930
Active travel	0.98	0.95,1.03	0.452	0.98	0.94,1.02	0.315	0.98	0.93,1.04	0.475
**Walk <30 mins/week**									
Urban/rural	0.99	0.93,1.06	0.788	0.97	0.91,1.04	0.450	0.91	0.82,1.02	0.099
NZdep	**1.09**	**1.05,1.14**	**<0.001**	**1.05**	**1.01,1.10**	**0.014**	**1.05**	**1.00,1.10**	**0.032**
Greenspace	**1.06**	**1.02,1.11**	**0.002**	**1.07**	**1.02,1.11**	**0.002**	**1.09**	**1.04,1.15**	**<0.001**
Foodshop	0.96	0.92,1.00	0.080	0.98	0.94,1.02	0.305	0.99	0.93,1.05	0.746
Gym/pool	1.02	0.98,1.06	0.391	1.01	0.97,1.05	0.776	1.00	0.94,1.06	0.960
Active travel	0.97	0.94,1.01	0.176	0.99	0.95,1.02	0.458	0.97	0.92,1.02	0.212
**Fruit/Veg 5/day**									
Urban/rural	**1.13**	**1.06,1.20**	**<0.001**	**1.09**	**1.03,1.17**	**0.004**	**1.18**	**1.06,1.32**	**0.003**
NZdep	**0.87**	**0.83,0.90**	**<0.001**	**0.94**	**0.90,0.99**	**0.011**	0.96	0.91,1.01	0.093
Greenspace	1.03	0.99,1.06	0.193	1.01	0.97,1.06	0.540	0.99	0.95,1.04	0.807
Foodshop	**1.10**	**1.05,1.14**	**<0.001**	1.04	1.00,1.09	0.056	1.03	0.97,1.10	0.331
Gym/pool	**1.05**	**1.01,1.09**	**0.015**	1.01	0.97,1.06	0.575	1.00	0.93,1.06	0.888
Active travel	0.98	0.94,1.01	0.211	1.00	0.96,1.04	0.892	1.03	0.98,1.09	0.236

Note: Categories: Urban/rural: 1 = Main urban area; 2 = Secondary urban area; 3 = Minor urban area; and 4 = Rural area. Environmental variables: quintiles (1 = best access, 5 = worst access); Deprivation (NZdep) (1 = least deprived, 5 = most deprived).

All bolded values are statistically significant at the 0.05 level.

Table 6 includes the results of regression analyses, where the ORs represent comparisons with the reference categories, for weight-related behaviours. Urban/rural status was significantly associated with meeting the recommended level of physical activity per week for the most rural category compared to the most urban category (OR = 1.47, p-value = 0.027). Living in neighbourhoods with low levels of active transport to work was, again surprisingly, associated with increased odds of being highly physically active. We found decreased odds of low levels of physical activity in areas of lower access to gym/pool compared to areas with high access, but the test of overall trend was not significant (p = 0.350). We found increased odds of walking 150 minutes or more per week for secondary urban areas compared to main urban areas (OR = 1.28, p = 0.016). We also found significant decreased odds of walking in the lowest categories of access to greenspace compared with the best level of access (ORs 0.73 and 0.76), with an overall significant trend (p <0.001). We also found significant associations between higher levels of deprivation and low levels of walking compared to the lowest deprivation category, with an overall significant trend (p = 0.032). Decreased access to greenspace was associated with increased odds of walking less than 30 minutes of walking per week, with an overall significant trend (p < 0.001). Last, we found that rural/urban status was associated with adequate consumption of fruits and vegetables, where more rural areas had increased odds of adequate consumption, compared to more urban areas, with an overall significant trend (p = 0.003).

**Table 6:** Association between obesity-related behaviours and environmental factors – fully adjusted models only

	**Category 1**	**Category 2**			**Category 3**			**Category 4**			**Category 5**			
		**OR**	**95% CI**	**p-value**	**OR**	**95% CI**	**p-value**	**OR**	**95% CI**	**p-value**	**OR**	**95% CI**	**p-value**	**p trend**
**Active 5+ days**														
Urban/rural	*Reference*	1.21	0.97,1.50	0.089	1.08	0.78,1.51	0.634	**1.47**	**1.05,2.06**	**0.027**				**0.038**
NZdep	*Reference*	0.97	0.81,1.16	0.717	0.92	0.75,1.12	0.380	1.00	0.83,1.20	0.983	1.10	0.89,1.36	0.364	0.113
Greenspace	*Reference*	1.06	0.89,1.26	0.913	1.15	0.97,1.38	0.082	0.93	0.78,1.11	0.316	0.94	0.75,1.18	0.598	0.579
Foodshop	*Reference*	1.13	0.95,1.34	0.159	0.96	0.81,1.14	0.612	1.09	0.88,1.34	0.451	1.34	0.98,1.83	0.064	0.599
Gym/pool	*Reference*	0.97	0.82,1.15	0.714	0.88	0.72,1.06	0.165	1.01	0.79,1.30	0.928	1.13	0.85,1.49	0.398	0.942
Active travel	*Reference*	0.84	0.70,1.01	0.067	**0.81**	**0.66,1.00**	**0.047**	**0.80**	**0.66,0.97**	**0.026**	**0.73**	**0.59,0.91**	**0.005**	**0.012**
**Active <30 mins/week**														
Urban/rural	*Reference*	1.14	0.81,1.59	0.458	1.16	0.61,2.21	0.641	1.13	0.62,2.06	0.681				0.548
NZdep	*Reference*	1.21	0.91,1.61	0.184	1.28	0.96,1.72	0.098	1.35	1.00,1.82	0.051	1.35	1.00,1.80	0.053	0.051
Greenspace	*Reference*	1.11	0.86,1.43	0.633	1.17	0.80,1.37	0.875	1.17	0.89,1.54	0.865	1.08	0.75,1.54	0.687	0.473
Foodshop	*Reference*	1.02	0.79,1.31	0.893	1.06	0.84,1.35	0.610	0.95	0.70,1.29	0.755	0.88	0.49,1.59	0.680	0.887
Gym/pool	*Reference*	0.92	0.72,1.18	0.501	0.98	0.76,1.25	0.846	**0.67**	**0.46,0.99**	**0.045**	0.85	0.56,1.30	0.460	0.350
Active travel	*Reference*	1.05	0.77,1.43	0.762	1.07	0.76,1.50	0.705	1.12	0.80,1.55	0.507	1.04	0.73,1.49	0.812	0.726
**Walk 150+ mins/week**														
Urban/rural	*Reference*	**1.28**	**1.05,1.57**	**0.016**	0.96	0.73,1.26	0.771	1.17	0.83,1.64	0.368				0.284
NZdep	*Reference*	1.07	0.87,1.30	0.529	1.01	0.83,1.24	0.898	1.11	0.91,1.36	0.315	1.15	0.92,1.43	0.222	0.208
Greenspace	*Reference*	1.05	0.88,1.25	0.677	0.94	0.78,1.14	0.600	**0.73**	**0.61,0.88**	**0.004**	**0.76**	**0.62,0.94**	**0.011**	**<0.001**
Foodshop	*Reference*	1.07	0.89,1.28	0.673	0.94	0.78,1.12	0.494	1.05	0.85,1.31	0.650	**1.36**	**1.00,1.83**	**0.048**	0.625
Gym/pool	*Reference*	0.95	0.79,1.14	0.564	0.88	0.72,1.07	0.195	1.06	0.83,1.36	0.635	1.07	0.80,1.43	0.642	0.930
Active travel	*Reference*	1.06	0.86,1.30	0.593	0.85	0.67,1.07	0.162	0.92	0.74,1.16	0.490	0.95	0.74,1.22	0.694	0.475
**Walk <30 mins/week**														
Urban/rural	*Reference*	0.96	0.78,1.18	0.697	0.84	0.62,1.14	0.260	0.74	0.51,1.06	0.096				0.099
NZdep	*Reference*	1.13	0.93,1.36	0.222	**1.39**	**1.14,1.70**	**0.001**	**1.26**	**1.03,1.54**	**0.022**	1.22	0.99,1.49	0.062	**0.032**
Greenspace	*Reference*	1.09	0.92,1.29	0.981	**1.24**	**1.03,1.49**	**0.026**	**1.37**	**1.14,1.64**	**0.001**	**1.36**	**1.09,1.70**	**0.006**	**<0.001**
Foodshop	*Reference*	0.90	0.76,1.07	0.230	0.91	0.77,1.09	0.311	0.94	0.76,1.17	0.583	1.05	0.77,1.43	0.768	0.746
Gym/pool	*Reference*	0.93	0.78,1.10	0.378	1.03	0.85,1.24	0.785	0.96	0.75,1.22	0.717	0.93	0.71,1.23	0.631	0.960
Active travel	*Reference*	1.00	0.82,1.23	0.976	1.11	0.89,1.38	0.368	1.03	0.82,1.28	0.818	0.86	0.67,1.09	0.213	0.212
**Fruit/Veg 5/day**														
Urban/rural	*Reference*	1.09	0.89,1.33	0.396	**1.45**	**1.05,2.02**	**0.025**	**1.68**	**1.19,2.36**	**0.003**				**0.003**
NZdep	*Reference*	1.04	0.86,1.25	0.705	0.86	0.72,1.05	0.134	1.00	0.82,1.24	0.930	**0.80**	**0.65,0.99**	**0.044**	0.093
Greenspace	*Reference*	1.06	0.88,1.27	0.413	1.04	0.86,1.25	0.429	1.03	0.86,1.23	0.336	0.94	0.75,1.19	0.636	0.807
Foodshop	*Reference*	1.12	0.93,1.35	0.222	1.10	0.91,1.33	0.339	1.19	0.95,1.49	0.125	0.98	0.70,1.39	0.922	0.331
Gym/pool	*Reference*	1.03	0.86,1.22	0.771	1.04	0.86,1.26	0.682	1.06	0.82,1.36	0.665	0.90	0.69,1.19	0.472	0.888
Active travel	*Reference*	0.99	0.82,1.20	0.950	1.13	0.92,1.40	0.242	1.18	0.96,1.46	0.123	1.08	0.86,1.36	0.497	0.236

Note: Categories: Urban/rural: 1 = Main urban area; 2 = Secondary urban area; 3= Minor urban area; and 4 = Rural area.

Environmental variables: quintiles (1 = best access, 5 = worst access); Deprivation (NZdep) (1= least deprived, 5= most deprived).

All bolded values are statistically significant at the 0.05 level.

## Discussion

We found that unhealthy weight categories and low levels of walking were all statistically significantly associated with area-level deprivation, independent of individual-level deprivation status, suggesting that overweight and obesity and walking are affected by neighbourhood context. Our findings are consistent with other New Zealand statistics indicating increased prevalence of obesity in more deprived neighbourhoods and [3] research which found that area-level deprivation was associated with obesity in adolescents [38]. These findings are consistent with conclusions from a number of countries including the UK [39], Australia [40] and the USA [41], which indicate the obesity and obesity-related behaviours was associated with neighbourhood deprivation, independent of individual income. Other New Zealand research has shown that other factors are correlated with neighbourhood deprivation which may affect walking behaviours, including both recorded and perceived crime [42]. The fear of neighbourhood crime has also exhibited a negative impact on mental and physical wellbeing in New Zealand [43], and has been shown to reduce residents’ walking within the local neighbourhood in Australia [44] and the UK [45]. Future research may further untangle the causal mechanisms in our identified association between overweight and obesity and walking behaviours and neighbourhood deprivation status by including neighbourhood crime measures.

Our results indicate that the local availability of greenspace promotes both healthy weight status and patterns of behaviour that facilitate the maintenance of healthy weight, particularly walking. Our findings are consistent with similar studies in England, which found proximity to greenspace to be protective against overweight and obesity and to promote physical activity [46]. However, in other New Zealand research using the same survey as our study and adjusting for similar individual-level confounders, Richardson et al found that proportion of greenspace within the neighbourhood was not related to overweight status [47]. However, in that study, several differences in variable measurement may have led to the differences in the observed relationships. First, our greenspace measures were proportions of useable greenspace as quintiles per meshblock unit, while Richardson et al calculated proportions of all greenspace per census area unit as quartiles. Second, Richardson et al restricted the sample to urban residents only. Last, we included individual-level confounders, area-level deprivation and all other environmental characteristics in our fully adjusted model. Yet, Richardson et al omitted area-level deprivation, urban/rural status, access to foodshop, access to gym and neighbourhood active travel. The inconsistent findings between these two studies highlight the importance of environmental variable measurement and inclusion variables. Similarly, we did not detect a significant association between access to greenspace and level of overall physical activity, but did find that increased access to greenspace was associated with higher levels of walking. However, Richardson et al found that individuals living in the greenest areas were significantly more likely to conduct at least 150 minutes of physical activity per week than those in the least green areas.

We also found that meeting the recommended level of physical activity per week was associated with urban/rural status, with higher activity in the more rural areas. This may relate to the trend for people to engage in physically active employment in rural areas, compared to urban areas. For example, one study found that individuals in China, Fiji and Malaysia living in urban areas were more physically active during leisure time but less active at work and in commuting compared to those in rural areas [48]. However, research from the US indicated that urban/rural status was not associated with pedometer-measured physical activity among adults [49].

Surprisingly, we also found a negative association between physical activity levels and percentage of neighbourhood residents engaging in active travel to work. The reasons for these findings are speculative at this stage. However, possible reasons include the tendency for areas of high active travel to be urban environments [50] with higher levels of traffic, pollution, and decreased safety. As such, engagement in physical activity (for leisure) may be less attractive. Comparison cannot be made with other international studies, as research in this area is scant most likely due to the lack of national data on neighbourhood commuting patterns which is available in New Zealand.

Results for adequate fruit and vegetable consumption show a significant urban/rural gradient, with more people meeting recommended levels in more rural compared to more urban areas. Possible reasons for this finding include the higher availability of home grown produce or availability of farm stands selling local produce. A recent review identified two USA studies which also found that residing in a rural area was associated with higher fruit and vegetable intakes [51]. A number of studies use distance to nearest supermarket, which could be considered a proxy for rural/urban status, to test the relationship with individual-level consumption of fruit and vegetable. In New Zealand, such a study found that geographic access to supermarkets was not associated with individuals’ vegetable intake [22]. Since our analyses adjusted for distance to nearest food shop, we identified an independent effect of urban/rural status on fruit/vegetable consumption.

This study is not without its limitations. The health and behaviour data used in this study were cross-sectional, limiting the ability to draw conclusions about causality. In addition, we did not account for the potential lag between exposure and outcome or the length of residence in a neighbourhood. Future longitudinal research could improve upon both of these limitations. In addition, all weight-related behaviours were self-reported. The use of objective measures of fruit/vegetable intake and physical activity would serve as future research improvements. Also, we were limited by the number of environmental variables permitted by the Ministry of Health. As a result, some relevant neighbourhood characteristics (e.g. crime rates) were not included and others were aggregated (e.g. foodshops included both grocery and fast food as one variable) in these analyses. The aggregation of types of food outlets limited our ability to separately assess the potential positive influence of access to stores offering healthy food options from the potential negative influence of access to unhealthy food options and therefore may be the reason for our largely null findings for this neighbourhood variable. Individuals may make use of several neighbourhood environments in addition to a residential neighbourhood. For example, individuals may exercise at a park near work or meet a friend in their neighbourhood for a walk. As such, expansion of environmental exposures to incorporate non-residential environments and percentage of time spent in each area would progress our understanding of the environmental determinants of this global epidemic, obesity.

## Conclusion

These results highlight greenspace as an amenable environmental factor associated with obesity/overweight. Park creation and planting in existing public spaces may serve as low-cost disease prevention options. Our results also indicate the potential benefit of targeted health promotion in both urban and deprived areas in New Zealand.

## Endnotes

^a^http://www.moh.govt.nz/moh.nsf/indexmh/portrait-of-health.

## Competing interests

We have no competing interests to declare.

## Authors’ contributions

All authors conceived of the study. PD performed data compilation and preliminary analyses. GB performed data analysis. AP drafted the manuscript. All authors provided edits to the manuscript. All authors read and approved the final manuscript.

## Pre-publication history

The pre-publication history for this paper can be accessed here:

http://www.biomedcentral.com/1471-2458/14/553/prepub
